# Stretchable h‐BN/TPU Fibers for Passive‐Active Dual‐Mode Thermal Management and Wearable Haptic Interfaces

**DOI:** 10.1002/advs.78162

**Published:** 2026-10-15

**Authors:** Yiwei Zhou, Shanshan Long, Bo Chen, Maohua Li, Linhong Li, Yue Qin, Rongjie Yang, Hanxi Chen, Cheng‐Te Lin, Yixiang Xu, Kean How Cheah, Nan Jiang, Jinhong Yu

**Affiliations:** ^1^ State Key Laboratory of Advanced Marine Materials Ningbo Institute of Materials Technology and Engineering Chinese Academy of Sciences Ningbo China; ^2^ Department of Mechancial Materials and Manufacturing Engineering Faculty of Science and Engineering University of Nottingham Ningbo China; ^3^ Center of Materials Science and Optoelectronics Engineering University of Chinese Academy of Sciences Beijing China

**Keywords:** hexagonal boron nitride, microfluidic spinning, stretchable composite fiber, thermal haptic interface, wearable thermal management

## Abstract

Wearable thermal interfaces require rapid, spatially confined thermal feedback together with mechanical compliance, yet existing passive thermal‐management textiles and active thermal‐haptic devices remain largely separate. Here, a stretchable *h*‐BN/TPU composite fiber is developed by coupling microfluidic spinning with non‐solvent‐induced phase separation. Rectangular‐channel confinement aligns hexagonal boron nitride plates within a microporous thermoplastic polyurethane matrix, constructing directional heat‐conduction pathways while preserving large deformation. The optimized 30 wt.% *h*‐BN/TPU fiber achieves a dry‐state thermal conductivity of 2.08 and 4.14 W·m^−1^·K^−1^ under humid conditions, together with 9.67 MPa tensile strength and 422% elongation at break. Pareto‐front analysis identifies the 30 wt.% composition as the thermal‐mechanical knee, arising from the coupled effects of filler orientation, low void fraction, and deformable TPU domains. Woven *h*‐BN/TPU fabrics provide passive thermal management, reducing surface temperature by 5.7°C under simulated solar irradiation and maintaining performance after 2000 bending cycles. When integrated into a thermal feedback glove, the fabric enables localized fingertip actuation with a heating rate of +29.55°C·s^−1^ and a thermal relaxation time of ∼20 s (returning below the warm perception threshold of 35°C). This fiber platform bridges passive heat regulation and active thermal haptics for next‐generation thermally interactive wearables.

## Graphical abstract

Optimized 30 wt.% *h*‐BN/TPU fibers create a thermal conductivity of 2.08 W·m^−1^·K^−1^ while retaining mechanical compliance (422% elongation at break). Woven fabrics reduce surface temperature by 5.7°C under simulated solar irradiation. Glove integration enables localized fingertip heating at 29.55°C·s^−1^ with a thermal relaxation time of approximately 20 s.

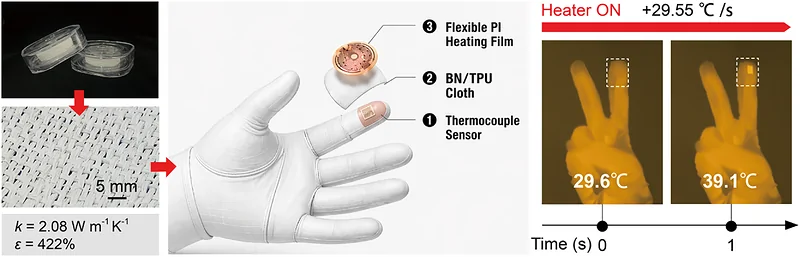

## 1 Introduction

Thermal sensation at the skin‐object interface is governed by thermal effusivity, interfacial contact resistance, and transient heat exchange, which together determine the temperature trajectory sensed by cutaneous thermoreceptors [1, 2, 3, 4]. Flexible thermal‐management textiles have been explored to regulate heat exchange in wearable electronics and personal thermal environments [5, 6, 7]. In parallel, fabric‐ and glove‐based thermal haptic devices have been developed for virtual reality and teleoperation, where localized heating and cooling can transmit material identity, environmental context, and affective information [7, 8, 9, 10, 11, 12]. Delivering rapid, spatially confined thermal stimulation while simultaneously preserving mechanical compliance, skin conformity, and ambient thermal comfort therefore imposes stringent and often conflicting requirements on the underlying material platform.

Existing platforms struggle to reconcile these requirements. Polymer‐based textile and glove substrates provide mechanical compliance and comfort but are intrinsically thermally insulating (*k* ≈ 0.1–0.3 W·m^−1^·K^−1^), which slows heat delivery and broadens the actuation area [5, 6]. Conductivity‐enhanced composites and thin conductive networks can overcome this heat‐transfer barrier, but high filler loading or electrically conductive pathways often compromise stretchability, breathability, or electrical isolation [13, 14, 15, 16, 17, 18, 19, 20, 21]. Active thermal glove systems based on pneumatic or thermoelectric actuation can generate perceivable thermal feedback, yet device‐level rigidity and multilayer integration can limit conformal skin contact under dynamic finger motion [7, 8, 12]. As a result, thermally functional textiles have evolved along two largely separate paths: passive thermal‐management fabrics deliver anisotropic heat dissipation but lack spatially programmable actuation, whereas active thermal‐haptic systems often rely on device architectures that reduce long‐term wearability. Bridging passive and active thermal functions within a single fiber‐based platform, while preserving fast response, spatial confinement, and large‐deformation tolerance, therefore remains an outstanding challenge.

Here we report a stretchable composite fiber platform that bridges passive thermal management and active, spatially confined thermal feedback within a single material system. Recent reviews have established next‐generation personal thermal management textiles around passive radiative/conductive heat dissipation, active heating/cooling, and adaptive moisture‐responsive thermoregulation, underscoring the growing demand for wearable systems that simultaneously manage thermal function and user comfort [21, 22, 23]. Anisotropic hexagonal boron nitride (h‐BN) plates are embedded within a thermoplastic polyurethane (TPU) matrix to construct continuous directional heat‐conduction pathways, exploiting the high in‐plane thermal conductivity and intrinsic electrical insulation of h‐BN [13, 24, 25, 26, 27, 28, 29, 30]. The dielectric character of h‐BN allows resistive heating elements and low‐voltage interconnects to be integrated directly within or adjacent to the fiber without an auxiliary insulating layer, while precluding short‐circuit pathways and inter‐element electrical crosstalk during repeated stretching of the wearable [18, 19, 20, 25, 31]. By contrast, electrically conductive fillers such as graphene, carbon nanotubes, or MXenes can introduce additional electrical‐isolation constraints in integrated wearable devices [13, 15, 16, 17]. Micron‐sized h‐BN plates are increasingly favored for constructing thermally conductive polymer composites [25, 26, 27, 28, 29, 30]. However, their rigid geometry and the high filler contents needed for network formation can disrupt the elastomeric matrix and degrade tensile behavior [26, 27, 28]. Aligned BN‐based thermal‐regulation textiles have demonstrated the value of anisotropic heat spreading for personal cooling, while thermo‐haptic reviews further highlight the need for localized, compliant, and spatially confined thermal feedback in wearable VR/AR interfaces [32, 33, 34]. We circumvent this trade‐off by combining microfluidic spinning with non‐solvent‐induced phase separation (NIPS). The flow‐driven elongation aligns h‐BN plates along the fiber axis, while phase separation generates a uniform microporous architecture within the fiber interior. The resulting hierarchical structure gives the micropores a coupled mechanical‐thermal role. They buffer local strain, preserving the large elongation of the TPU matrix despite the rigid h‐BN loading. The interconnected pore channels also admit ambient moisture into the fiber interior, where absorbed water replaces insulating air within the pore space and likely improves local thermal coupling between nearby aligned h‐BN plates under humid conditions. In the dry state, the same air‐filled pores instead suppress through‐plane heat transport, so that thermal stimuli remain laterally confined at the skin interface rather than diffusing into surrounding fabric areas.

By exploiting this multifunctionality, the 30 wt.% h‐BN/TPU fiber serves two complementary roles—woven into a fabric for passive heat regulation, and integrated with heating elements for localized thermal feedback—realizing dual‐mode thermally interactive wearables from a single fiber chemistry. Notably, the aligned‐h‐BN/microporous‐TPU architecture reaches a thermal conductivity an order of magnitude above non‐oriented TPU fibers while retaining the large elongation of the elastomeric matrix, a combination seldom achieved by highly loaded thermally conductive fibers. By unifying aligned *h*‐BN plates, a microporous architecture, and an elastomeric TPU matrix within a single fiber, this work establishes a multifunctional material platform that integrates directional thermal conduction, mechanical compliance, moisture‐responsive thermal enhancement, and dual‐mode passive–active thermal regulation. The fiber form factor is intrinsically compatible with standard textile manufacturing—weaving, knitting, and embroidery—and supports seamless integration with active heating elements, providing a scalable route toward wearable thermal interfaces for virtual and augmented reality, teleoperation, biomedical rehabilitation, and adaptive personal thermal management. At the textile level, future control over fiber layout, such as knitted loops or multi‐angle arrangements, may provide an additional route to tune stretchability, conformal contact, and in‐plane heat spreading without changing the single‐fiber composition.

## 2 Results and Discussion

### 2.1 Preparation Process

The fiber design followed a coupled structure–processing rationale rather than empirical formulation screening alone, integrating filler selection, channel‐confined alignment, and phase‐separation‐induced porosity into a single spinning process. Figure 1a illustrates the continuous microfluidic spinning process used to fabricate the h‐BN/TPU fibers. A h‐BN/TPU/DMF spinning dope is extruded through a rectangular microchannel (slit geometry) before entering a water coagulation bath. Within the channel, geometric confinement converts the incoming circular flow into a slit‐shaped flow field and imposes wall‐confined shear on the anisotropic h‐BN plates, reducing their rotational freedom and biasing the high‐aspect‐ratio platelets into an axially aligned state; non‐solvent‐induced phase separation (NIPS) in the subsequent water bath then fixes this oriented morphology (Figure 1b).

**FIGURE 1:**
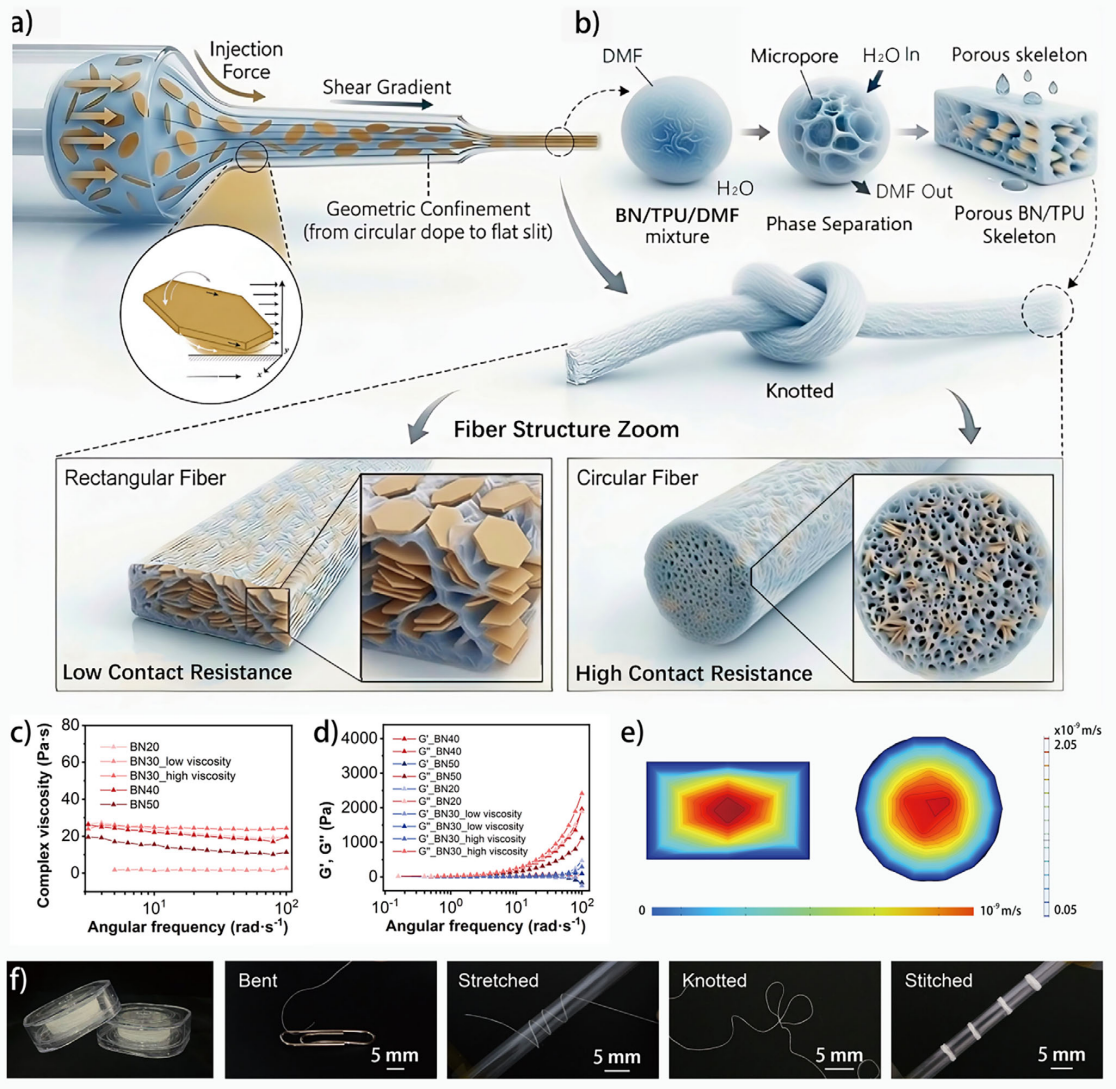
Fabrication and structural design of rectangular h‐BN/TPU fibers. (a) Schematic of continuous microfluidic spinning in a rectangular channel. (b) Schematic of non‐solvent‐induced phase separation (NIPS) during fiber formation. (c,d) Complex viscosity and viscoelastic moduli of the spinning dopes. (e) Simulated shear fields in rectangular and cylindrical channels. (f) Photographs of the fibers during bending, stretching, knotting, and textile integration (scale bars, 5 mm).

This channel design was guided by the inertial focusing behavior summarized in Figure S1, in which the competition between wall‐effect lifts and shear‐gradient lift produces stable off‐center equilibrium positions in confined channels, with rectangular geometries favoring positions near the wider channel faces [29, 30, 35, 36, 37]. We invoke this confinement principle qualitatively: under the combined influence of shear‐induced migration, wall confinement, and flow alignment, the 2D h‐BN plates are expected to migrate toward the channel walls and orient parallel to the wall surfaces, providing a structural basis for continuous anisotropic thermal conduction paths along the fiber axis (*x*‐axis) [20, 29, 38].

Large‐diameter, highly crystalline h‐BN plates (57 µm, Figure S3) were selected because larger oriented plates can reduce filler‐filler junction density and cumulative interfacial thermal resistance in h‐BN polymer composites [39, 40]. These design principles build on an earlier demonstration of uniaxially oriented large‐plate h‐BN composites for wireless‐charging thermal management, where dense laminated structures achieved ultra‐high through‐plane conductivity but precluded textile‐compatible deformability [41]. The present work transfers the orientation‐and‐interface logic of that platform from a dense laminate to a stretchable fiber form, introducing controlled porosity as an additional design lever. Within the customized rectangular focusing channel (width 200 µm, aspect ratio 2.5), shear‐induced alignment under flow confinement and the viscoelastic behavior of TPU governs filler orientation. The channel dimensions can restrict plate rotation, enhance shear anisotropy, and maintain stable extrusion without clogging, enabling a highly aligned filler microstructure along the fiber axis [42]. More broadly, flow‐field engineering has been shown to rotate BN platelets and tailor anisotropic thermal pathways in polymer composites, supporting microchannel‐guided alignment as a rational, rather than empirical, design strategy [27, 34].

Meanwhile, the incorporation of rigid inorganic fillers is known to disrupt polymer‐chain mobility and potentially compromise fiber stretchability [43, 44]. To address this issue at the structural‐design level, a wet‐spinning strategy was proposed to introduce uniformly distributed micropores inside the fibers (Figure 1b). These micropores are expected to act as deformable structural units that facilitate energy dissipation during tensile deformation and alleviate filler‐induced stress concentration [45, 46, 47]. Therefore, the combined design of filler alignment and porous fiber architecture is intended to improve thermal conduction while preserving mechanical compliance in *h*‐BN/TPU fibers.

Rheological characterization (Figure 1c,d and Note S1) was conducted to evaluate the shear‐dependent flow behavior and viscoelastic properties of the *h*‐BN/TPU spinning dopes during microfluidic extrusion. The viscosity curves reveal pronounced shear‐thinning, indicating that apparent viscosity decreases with increasing shear, which facilitates dope deformation and continuous flow within the rectangular microchannel. Oscillatory measurements further confirm a viscoelastic liquid state, with the loss modulus (*G″*) exceeding the storage modulus (*G′*). Increasing *h*‐BN content leads to higher *G′* and *G″* and a narrowed gap between the two moduli, consistent with the formation of a progressively interconnected filler network that can constrain polymer‐chain mobility while providing structural support.

Finite element simulations (Figure 1e and Figure S5) show that, under identical flow rates, the slit‐shaped channel generates an extended, directionally biased high‐velocity region compared with a circular channel. When combined with the shear‐thinning behavior of the spinning dopes, this flow profile enhances wall‐constrained shear and geometric guidance, promoting near‐wall migration and directional alignment of *h*‐BN plates. Together, the rheological properties and flow‐field distribution provide a mechanistic basis for both the facile deformation of the dope and the formation of continuous, aligned thermal pathways in the resulting fibers, while highlighting the need for a porous TPU framework to accommodate strain and dissipate tensile energy.

After the flow‐induced alignment within the rectangular microchannel, the wet‐spinning coagulation process was designed to further preserve the oriented *h*‐BN configuration while constructing a porous TPU framework. Upon extrusion into the water bath, solvent exchange between N,N‐dimethylformamide (DMF) and water triggers non‐solvent‐induced phase separation, leading to the formation of uniformly distributed micropores within the TPU matrix (Figure 1b) [39, 48, 49]. Meanwhile, the air‐water interface during fiber formation may provide an additional interfacial constraint that assists the parallel arrangement of *h*‐BN plates near the fiber surface. This combined microchannel‐guided alignment and phase‐separation‐induced porous architecture is expected to provide a structural basis for axial heat transport while introducing deformable units for strain buffering and energy dissipation.

The wet‐spinning strategy further enables continuous fabrication of *h*‐BN/TPU fibers with diameters of approximately 150–200 µm. The obtained fibers can be bent, stretched, and knotted without obvious structural failure (Figure 1f), demonstrating their potential compatibility with flexible and wearable thermal management systems.

## 3 Structural Characterization

To validate the geometric confinement effect proposed in our design strategy, the internal microstructures of *h*‐BN/TPU fibers fabricated using different channel geometries were first compared. As shown in Figure 2a, the cross‐sectional SEM image of a representative 30 wt.% *h*‐BN/TPU fiber produced through a conventional cylindrical channel shows a random and disordered distribution of *h*‐BN plates. Without lateral geometric confinement, the high‐aspect‐ratio *h*‐BN plates undergo less restricted rotational motion under shear flow, leading to crossed and stochastic arrangements. This result indicates that conventional cylindrical extrusion provides limited control over filler orientation during fiber formation.

**FIGURE 2:**
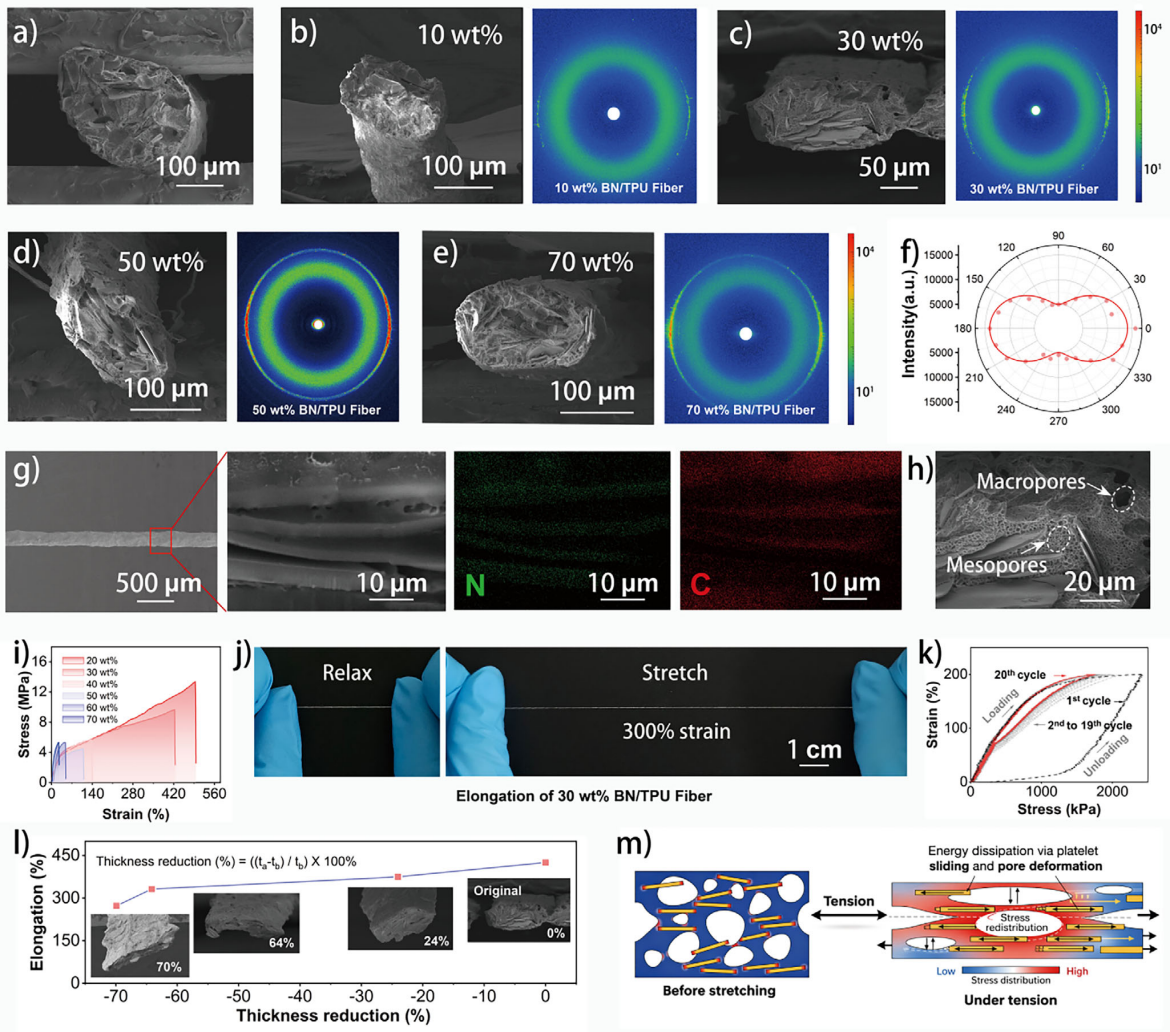
Structural characterization and stretchability of h‐BN/TPU fibers. (a) Cross‐sectional SEM image of a 30 wt.% h‐BN/TPU fiber spun in a cylindrical channel. (b–e) Cross‐sectional SEM images and 2D‐WAXRD patterns of 10 wt.% (b), 30 wt.% (c), 50 wt.% (d), and 70 wt.% (e) fibers. (f) Angle‐resolved polarized Raman response and fitting curve. g) FIB‐SEM and EDS (N, C) images of the 30 wt.% h‐BN/TPU fiber sectioned along the *z*‐axis. (h) Porous structure of the 30 wt.% h‐BN/TPU fiber. (i) Mechanical performance versus h‐BN content (20–70 wt.%). (j) Cyclic tensile loading‐unloading curves at 200% strain for 20 cycles. (k) Photograph of the 30 wt.% h‐BN/TPU fiber stretched to 300% strain. (l) Relationship between porous content and tensile elongation. m) Schematic of energy dissipation through platelet sliding and pore deformation.

In contrast, fibers fabricated using the customized rectangular focusing channel exhibit a distinctly more ordered internal morphology. The *h*‐BN plates are preferentially arranged parallel to the channel walls, with their long axes oriented along the y direction, providing a structural basis for anisotropic thermal conduction. To further examine the effect of filler concentration under this confined flow field, *h*‐BN/TPU fibers with *h*‐BN loadings from 10 to 70 wt.% were systematically investigated (Figure 2b–e). SEM observations show that increasing *h*‐BN loading gradually leads to a denser and more ordered plate arrangement. This structural evolution is attributed to the combined effects of the pronounced shear gradient generated within the rectangular focusing channel and the spatial confinement that restricts plate rotation. The preferential orientation of h‐BN plates was further verified using 2D‐wide‐angle x‐ray diffraction (2D‐WAXRD). With increasing h‐BN content, the diffraction rings corresponding to the h‐BN (002) planes gradually transform from diffuse halos into concentrated equatorial arcs, indicating enhanced plate alignment. In the coordinate system used throughout this work, the *x*‐axis denotes the fiber axial direction, while the y‐ and z‐axes span the transverse cross‐sectional plane. For the aligned fibers, the (002) equatorial arcs therefore indicate that the h‐BN basal planes preferentially align parallel to the fiber axis, whereas their platelet normals are oriented predominantly in the transverse plane. Quantitative analysis of the azimuthal intensity distribution gives a Hermans orientation factor of f = 0.72 for the 30 wt.% h‐BN/TPU fiber. In this definition, f = 1 corresponds to platelet normals fully perpendicular to the fiber axis (that is, ideal axial alignment of the h‐BN basal planes), whereas f = 0 represents an isotropic orientation state. This value therefore confirms a high degree of structural anisotropy compared with isotropic composites. The detailed calculation process is provided in Note S2. Polarized Raman spectroscopy provides additional evidence for the preferential filler orientation. As shown in Figure 2f, the intensity of the characteristic E2g mode of h‐BN at 1360 cm^−1^ varies with the polarization direction of the incident laser and reaches its maximum when the polarization direction is parallel to the fiber axis. The resulting two‐lobed polar pattern further indicates the preferential alignment of h‐BN plates within the TPU matrix. The detailed analysis is provided in Note S3. Consistent h‐BN alignment along the fiber direction is also confirmed by SEM and corresponding EDS mapping of the 30 wt.% h‐BN/TPU fiber (Figure 2g), where the nitrogen and carbon elemental maps clearly reveal the parallel arrangement of h‐BN plates within the TPU matrix.

Structural characterization also confirms that NIPS produced an internal pore hierarchy rather than a dense filler‐filled strand. In the 30 wt.% h‐BN/TPU fiber (Figure 2h), mesopores of 1.2–2.2 µm coexist with macropores of 8.9–9.4 µm, with mesopores dominating the overall porous structure. This observation turns the design premise in Figure 1b into direct microstructural evidence: aligned h‐BN plates are embedded in a deformable porous TPU scaffold, setting up the subsequent mechanical tests of pore‐assisted strain accommodation.

To evaluate the suitability of the fibers for stretchable wearable applications, the tensile behavior was systematically investigated as a function of *h*‐BN content. Although increasing *h*‐BN loading can improve filler connectivity, excessive rigid fillers may disrupt the deformable TPU network and intensify interfacial stress concentration. As shown in Figure 2i, the mechanical performance exhibits a non‐monotonic dependence on *h*‐BN loading: at a moderate loading of 30 wt.%, the single *h*‐BN/TPU fiber still retains a high elongation at break of approximately 422%, whereas the tensile deformability deteriorates significantly when the *h*‐BN content exceeds 40 wt.%. This degradation is attributed to increased structural defects, filler aggregation, and enhanced stress concentration at high filler loadings.

Based on this thermal‐mechanical trade‐off, 30 wt.% *h*‐BN was identified as the optimal composition for achieving balanced stretchability and thermal performance. The high elongation capability of the 30 wt.% *h*‐BN/TPU fiber is demonstrated in Figure 2k, while cyclic tensile testing up to 200% strain over 20 cycles further confirm its deformation tolerance and mechanical durability. As shown in Figure 2j, the first loading‐unloading cycle exhibits a pronounced hysteresis loop, indicating substantial energy dissipation during the initial deformation.

Consequently, the mechanical comfort of the 30 wt.% *h*‐BN/TPU fiber‐based fabric can be evidenced by its exceptional deformability and cyclic stability. The fibers exhibit a fracture strain exceeding 420% and a low initial modulus, accommodating substantial hand deformations. Cyclic loading‐unloading tests show that under typical operational strains (e.g., 50%), the induced tensile stress is approximately 0.3 MPa, within the lower range of human tactile perception (∼0.2–0.76 MPa per fiber, based on prior studies [40, 50, 51, 52]), effectively eliminating noticeable resistance. After the first cycle, subsequent curves (2nd–19th) largely overlap, and the 20th cycle maintains a similar profile up to 200% strain without mechanical failure, demonstrating excellent cyclic stability and elastic recovery. Together, these results confirm that the fibers provide a flexible, unobtrusive, and comfortable user experience while sustaining repeated deformations.

The excellent tensile elongation at break arises from the rearrangement and interfacial sliding of *h*‐BN plates, viscoelastic deformation of the TPU matrix, partial deformation of the porous framework, and the formation of a *h*‐BN‐TPU interfacial layer that facilitates efficient stress transfer and local energy dissipation, analogous to interface effects observed in rubber‐filler nanocomposites. To clarify the role of the porous architecture, 30 wt.% *h*‐BN/TPU fibers were roll‐pressed with progressively reduced gaps. Cross‐sectional SEM and mechanical results (Figure 2l) show that elongation decreases as the pores collapse, highlighting the microporous framework's critical contribution to large deformation. This effect resembles that of interfacial polymer layers in polymer‐inorganic nanocomposites, where pores and interfaces collectively regulate stress distribution and local segmental mobility. Under tensile loading, the fiber's response reflects a synergistic interplay of three modes: extensible TPU chains accommodate strain, interfacial sliding between *h*‐BN plates redistributes stress, and micron‐scale pores elongate or collapse to dissipate energy. Filler size, alignment, and surface properties further modulate stress transfer and chain mobility, ultimately governing macroscopic mechanical performance.

In summary, the mechanical data narrow the design rule from a general filler‐loading problem to a structure‐controlled optimum. The 30 wt.% fiber preserves enough connected *h*‐BN contacts for heat transfer while retaining a pore‐enabled TPU scaffold for strain accommodation; increasing h‐BN beyond this window strengthens the filler network but consumes deformable matrix volume and amplifies stress localization. The structural and mechanical results therefore identify 30 wt.% as the composition where alignment, porosity, and elastomer continuity remain simultaneously active.

## 4 Thermal Properties

As shown in Figure 3a, from the perspective of the weaving process, rectangular fibers exhibit significantly larger inter‐fiber contact areas than conventional cylindrical fibers, which is expected to reduce contact thermal resistance and enhance in‐plane heat spreading in *h*‐BN/TPU fabrics. FEA were therefore conducted to compare heat transfer between aligned cylindrical and rectangular fiber assemblies. As shown in Figure 3b,c, under the simulated conditions, the rectangular‐fiber assembly exhibits more efficient heat transfer from the hot side to the cold side, with an equilibrium temperature difference of 20°C compared with the cylindrical‐fiber assembly while heated to 100°C. This result highlights the importance of fiber geometry in regulating fabric‐level thermal transport.

**FIGURE 3:**
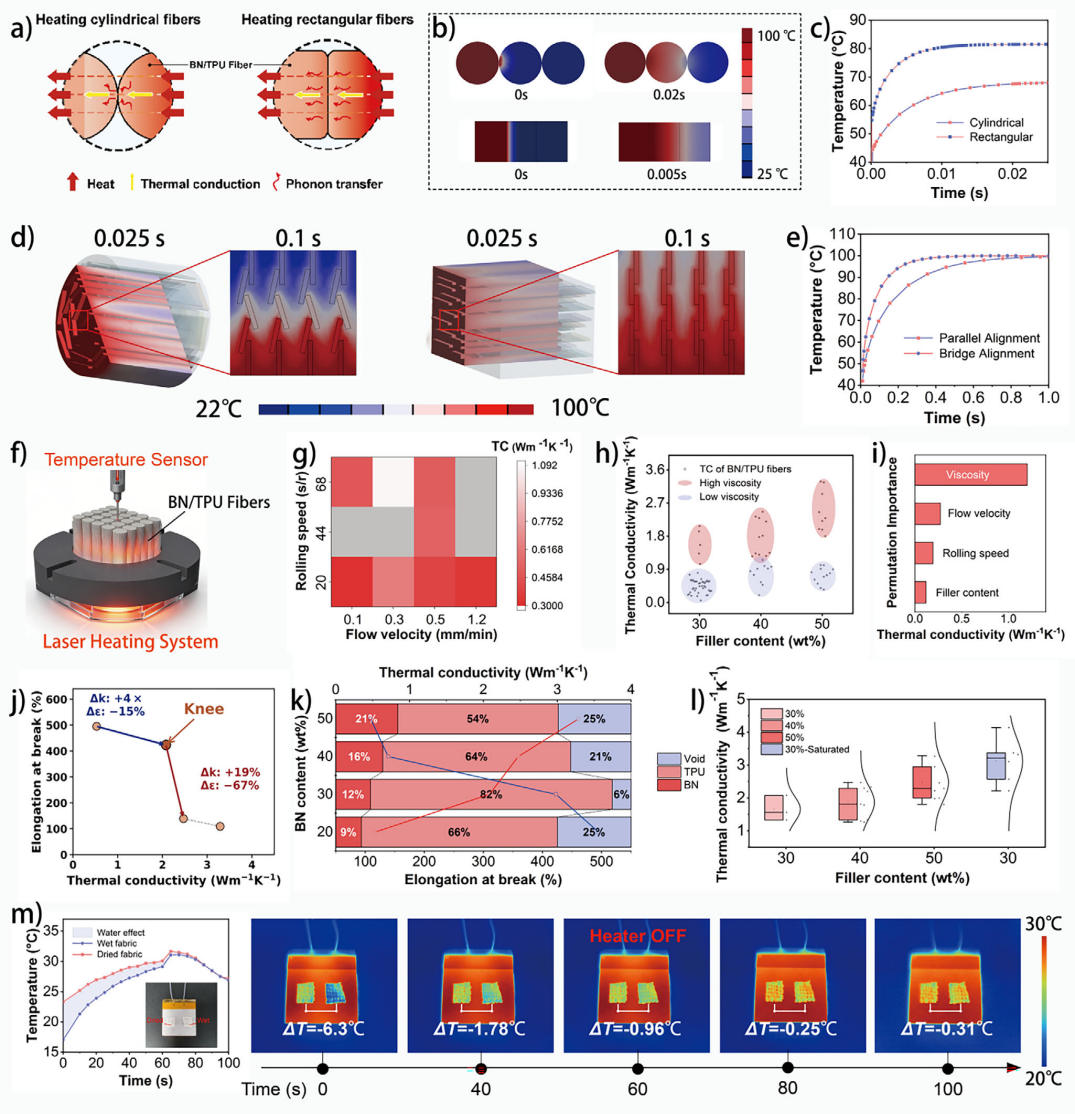
Thermal transport optimization and structure‐property analysis of h‐BN/TPU fibers. (a–c) Schematics comparing heat transfer in cylindrical and rectangular fiber assemblies. (d,e) Finite element analysis (FEA) of heat transfer for line‐face and face‐face h‐BN contact configurations. (f) Schematic of axial thermal conductivity measurement using bundled fibers. (g) Thermal conductivity map as a function of injection flow velocity and collector rolling speed under low‐viscosity conditions. (h) Dependence of thermal conductivity on solution viscosity at different h‐BN contents. (i) Random‐forest permutation‐importance ranking of the processing parameters. (j) Pareto‐front analysis of thermal conductivity and elongation at break. (k) Thermal conductivity and elongation at break in relation to the BN, TPU, and void fractions. (l) Thermal conductivity of fibers at different filler loadings under dry and humid conditions. (m) Infrared temperature‐time curves and images of dry and humid 30 wt.% fabrics.

At the filler‐network scale, thermal transport behavior was analyzed via FEA modeling, incorporating both matrix‐filler and filler‐filler interfacial thermal resistances. SEM observations reveal that fibers extruded through traditional cylindrical channels contain randomly oriented, interlaced *h*‐BN plates, resulting in line‐face contacts between adjacent plates (Figure 2a). In contrast, fibers produced through rectangular channels exhibit *h*‐BN plates aligned nearly parallel to the fiber axis (Figure 2c), facilitating predominantly face‐face contact topology. FEA simulations (Figure 3d,e and Figure S8) confirm that this face‐face contact topology provides larger planar overlap and lower effective filler‐filler interfacial resistance compared to the line‐face contact topology characteristic of cylindrical fibers. This microscale advantage, supported by the SEM evidence of parallel alignment within rectangular fibers, validates the geometric rationale presented in Figure 3b,c.

The axial thermal conductivity of bundled h‐BN/TPU fibers was then measured using the laser flash method (Figure 3f) to validate the trends suggested by FEA and SEM observations, where, specifically, individual fibers were assembled into aligned bundles to obtain a measurable specimen, and the thermal transport was probed along the fiber axial (z) direction. As shown in Figure 3g, the thermal conductivity along the x‐axis is strongly influenced by the injection flow rate and collector rolling speed, which synergistically affect *h*‐BN plate rearrangement and fiber formation during microfluidic spinning. Notably, analysis of Figure 3g indicates that an injection velocity of 0.3 mm/min yields the highest thermal conductivity, suggesting that this flow rate optimally promotes shear‐induced alignment of *h*‐BN plates along the fiber axis.

The influence of spinning dope viscosity is highlighted in Figure 3h. Increasing the viscosity of the h‐BN/TPU/DMF solution within a reasonable range (e.g., increasing to 8988 mPa·s from 1717 mPa·s) significantly enhances the fiber thermal conductivity. The observed thermal conductivity enhancement can be consistently attributed to stronger viscous stress transfer, reduced platelet rotational relaxation, and improved retention of shear‐induced axial alignment within the confined channel. Within the present processing window, these effects favor the formation of more continuous h‐BN heat‐conduction pathways along the fiber axis.

To further quantify the relative contributions of different processing parameters, a random forest model was employed to analyze the permutation importance of four factors: *h*‐BN content, solution viscosity, injection flow rate, and collector rolling speed (Figure 3i). The results indicate that, within the 30–50 wt.% *h*‐BN range, solution viscosity is the most critical factor controlling thermal conductivity during wet spinning, consistent with the shear‐alignment mechanism established from Figure 3g,h. In comparison, *h*‐BN content, often considered the dominant factor in thermally conductive composites, exerts a weaker effect under the present processing conditions with large *h*‐BN plates and confined microchannel flow. Details of the model implementation are provided in Note S5.

To evaluate the thermal‐mechanical trade‐off without imposing a priori weights between the two objectives, the four *h*‐BN/TPU fiber samples were first analyzed in the (*k*, *ε*) plane (Figure 3j). All four compositions lie on the Pareto front, with none dominated by another. The trade‐off rate along this front is strongly composition‐dependent: from 20 to 30 wt.% *h*‐BN, axial thermal conductivity rises by 4× while elongation decreases by only 15%, whereas from 30 to 40 wt.% *h*‐BN, *k* gains less than 20% at the cost of a 67% drop in elongation. The 30 wt.% composition thus lies at the knee of the Pareto front, where each unit of additional *k* requires a disproportionately small *ε* sacrifice. For a compact figure‐of‐merit comparison across compositions, a thermal‐mechanical balance factor (BF) was also computed:

(1) $$\mathrm{BF}=(\varepsilon_{\mathrm{fiber}}\;/\;\varepsilon_{\max})\;\cdot \;(k_{\mathrm{fiber}}\;/\;k_{\max})$$

where *ε_fiber_* and *k_fiber_* represent the elongation at break and axial thermal conductivity of each fiber, respectively, while *εmax* and *kmax* are the maximum values among the tested samples. The *BF* reaches its maximum of 0.63 at 30 wt.%, consistent with the Pareto‐knee position identified above. The remaining *BF* values across compositions are summarized in Table S1. This formulation assigns equal weight to thermal and mechanical contributions; for applications prioritizing one property over the other, a weighted product *w_1_(k/k_max_) + w_2_(ε/ε_max_)* can be substituted with application‐specific coefficients.

To uncover the structural origin of the Pareto‐knee position, the volume fractions of *h*‐BN, TPU, and voids were determined for the 30, 40, and 50 wt.% samples (Figure 3k). The 30 wt.% fiber contains a void fraction 3–4× lower than its 40 and 50 wt.% counterparts. This anomalously low porosity is the direct structural explanation for the knee: it minimizes air‐induced thermal resistance while leaving sufficient deformable TPU domains for strain accommodation, simultaneously preserving both *k* and ε.

The low void fraction of the 30 wt.% fiber is likely associated with the processing window established during microfluidic spinning and NIPS. At this composition, the spinning‐dope viscosity, injection speed, and collector rolling speed can jointly regulate solvent–non‐solvent exchange and matrix solidification, allowing the forming TPU network to densify before large voids are kinetically trapped. In contrast, higher *h*‐BN loadings may restrict polymer‐chain mobility and accelerate kinetic arrest during phase separation, preserving more voids and defects. This process‐enabled pore regulation explains why the 30 wt.% sample combines efficient thermal transport with high deformability, rather than behaving simply as an intermediate‐filler‐loading composition. Therefore, the 30 wt.% optimum should be understood as a composition–processing coupled window rather than a single‐variable filler‐loading effect. The h‐BN content provides sufficient platelet contacts and viscosity‐mediated alignment, while the relatively low injection velocity and appropriate collector rolling speed tune residence time, drawing, and solvent–non‐solvent exchange during coagulation.

To elucidate the role of porosity on the thermal conductivity of *h*‐BN/TPU composite fibers, the fiber thermal conductivity was analyzed using a single‐degree‐of‐freedom decoupled Lewis‐Nielsen model [53, 54]. The model was modified to incorporate decoupled pore suppression at low and high *h*‐BN content, as detailed in Note S4. In this approach, the thermal network is described as a three‐phase system comprising *h*‐BN plates, the TPU matrix, and air‐filled pores. The shape factor of *h*‐BN plates along the fiber axis was corrected using the Halpin‐Tsai approach [55]:

(2) $$A_{eff}=2D/t\cdot f_{H}+A_{random}\cdot (1-f_{H})$$

where D/t = 57 is the aspect ratio of the h‐BN plates, fH = 0.72 is the WAXRD‐derived Hermans orientation factor describing the degree to which the h‐BN basal planes align parallel to the fiber axis, and Arandom = 2.5 corresponds to randomly oriented plates. The maximum packing fraction of h‐BN was fixed at φm = 0.736, and the thermal conductivities of the individual components were set as kBN = 200 W·m^−1^·K^−1^, kTPU = 0.3 W·m^−1^·K^−1^, and kvoid = 0.025 W·m^−1^·K^−1^.

To translate this composition‐coupled behavior into a quantitative framework, the pore suppression factor *C_void_* in the Lewis‐Nielsen formulation was decoupled into two regimes: *C_low_* = 3.075 for low *h*‐BN content and *C_high_* = 1.0 for high *h*‐BN content. The substantially larger *C_low_* captures the high sensitivity of sparse *h*‐BN networks to void interruption; each pore directly truncates one of the few percolative filler‐filler contact paths available, producing a disproportionately large reduction in conductivity per unit void fraction. As *h*‐BN content increases, the network gains redundancy and pores are bypassed by alternative phonon pathways, so the void penalty asymptotes to the geometric spherical‐inclusion limit (*C_high_* ≈ 1.0). The 30 wt.% sample, which combines low *h*‐BN content with anomalously low void fraction, is the only composition that uniquely escapes the otherwise punitive *C_low_* regime.

Applying this decoupled model to the four experimental data points (Table S1) captures the transition from the island regime to the filler‐network regime. Within the network‐regime samples (S2‐S4), the parity plot between *k_exp_* and *k_fit_* gives *R^2^* = 0.986, indicating that the decoupled Lewis‐Nielsen treatment reproduces the experimental trend in this domain. The lowest‐loading sample (S1, *φ_BN_* = 0.087) falls outside the model's network‐regime domain and shows a systematic overestimation of 61%; we attribute this not to a fitting failure but to the sub‐percolation nature of this composition. At *φ_BN_* below the network‐formation threshold, *h*‐BN plates remain dispersed as isolated islands within the TPU matrix, and the effective‐medium assumption of additive filler contributions overestimates the thermal conduction path density. This deviation therefore provides independent evidence for the network‐to‐island transition near 30 wt.% *h*‐BN, the same structural transition that underlies the Pareto‐knee behavior identified in Figure 3j. Parameter stability across the explored *C_low_* and *C_high_* range was further verified via Monte Carlo simulations (Note S4) [56, 57, 58, 59, 60].

Subsequently, the physical significance of the fitted decoupled *C_void_* was independently verified by SEM imaging of the fiber cross‐sections. The fibers contain both macro‐ and mesopores, with macro‐pores elongated along the fiber axis and mesopores densely distributed, yielding an average micro‐pore aspect ratio of *L/W* ≈ 1.44 (Table S2). This anisotropy substantially increases the effective thermal resistance relative to idealized spherical pores, consistent with the fitted decoupled *C_void_* values. Smaller, densely distributed pores interrupt local heat paths, and pores are often located near *h*‐BN plates, supporting a coupling effect between pore morphology and *h*‐BN alignment. These observations establish a direct link between macro‐scale thermal conductivity and microstructural pore characteristics.

Apart from what was mentioned above, a notable feature of the porous h‐BN/TPU fibers is their humidity‐assisted thermal transport. Under humid conditions, the thermal conductivity of 30 wt.% h‐BN/TPU fibers nearly doubles (Figure 3l). Water uptake tests show that the 30 wt.% fibers can fill their internal micropores with water, achieving nearly full saturation of the pore volume, which corresponds to 28 wt.% relative to the total fiber mass. This enhancement of the overall thermal conductivity is primarily attributed to the replacement of thermally insulating air (0.025 W·m^−1^·K^−1^) within the micropores by absorbed water (0.6 W·m^−1^·K^−1^), which reduces pore‐associated thermal resistance. In addition, the absorbed water likely improves local thermal coupling between adjacent h‐BN plates across short pore gaps, but this interfacial contribution should be regarded as a plausible assisting mechanism rather than a directly resolved conduction pathway in the present study. Thus, the internal micropores play a dual role: in the dry state, a controlled porous structure supports mechanical energy dissipation, while under humid conditions, water‐filled pores can further enhance thermal transfer. The thermal behavior under humid conditions was then investigated using IR device (Figure 3m). A temperature difference of 6.3°C is observed between wet and dry fabrics at the beginning. After 1 min of heating, the internal moisture evaporates, as evidenced by the convergence of the temperature curves following the heater OFF event. This behavior indicates rapid moisture dissipation, which can be attributed to the synergistic effects of efficient in‐plane heat conduction through aligned h‐BN networks and the intrinsic air permeability of the h‐BN/TPU fabric.

## 5 Application

As illustrated in Figure 4a,b, the 30 wt.% *h*‐BN/TPU fabric forms the core material of a thermal feedback glove for VR applications. The dielectric character of *h*‐BN/TPU allows the PI heater to be laminated directly onto the fabric without an additional insulating layer, simplifying device architecture while maintaining thin form factor. The fabric exploits in‐plane (high) and through‐plane (moderate) thermal conductivity anisotropy, modulating heat delivery to the skin: rapid lateral spreading ensures uniform actuation while through‐plane resistance prevents excessive local heating, avoiding perceptual discontinuities.

**FIGURE 4:**
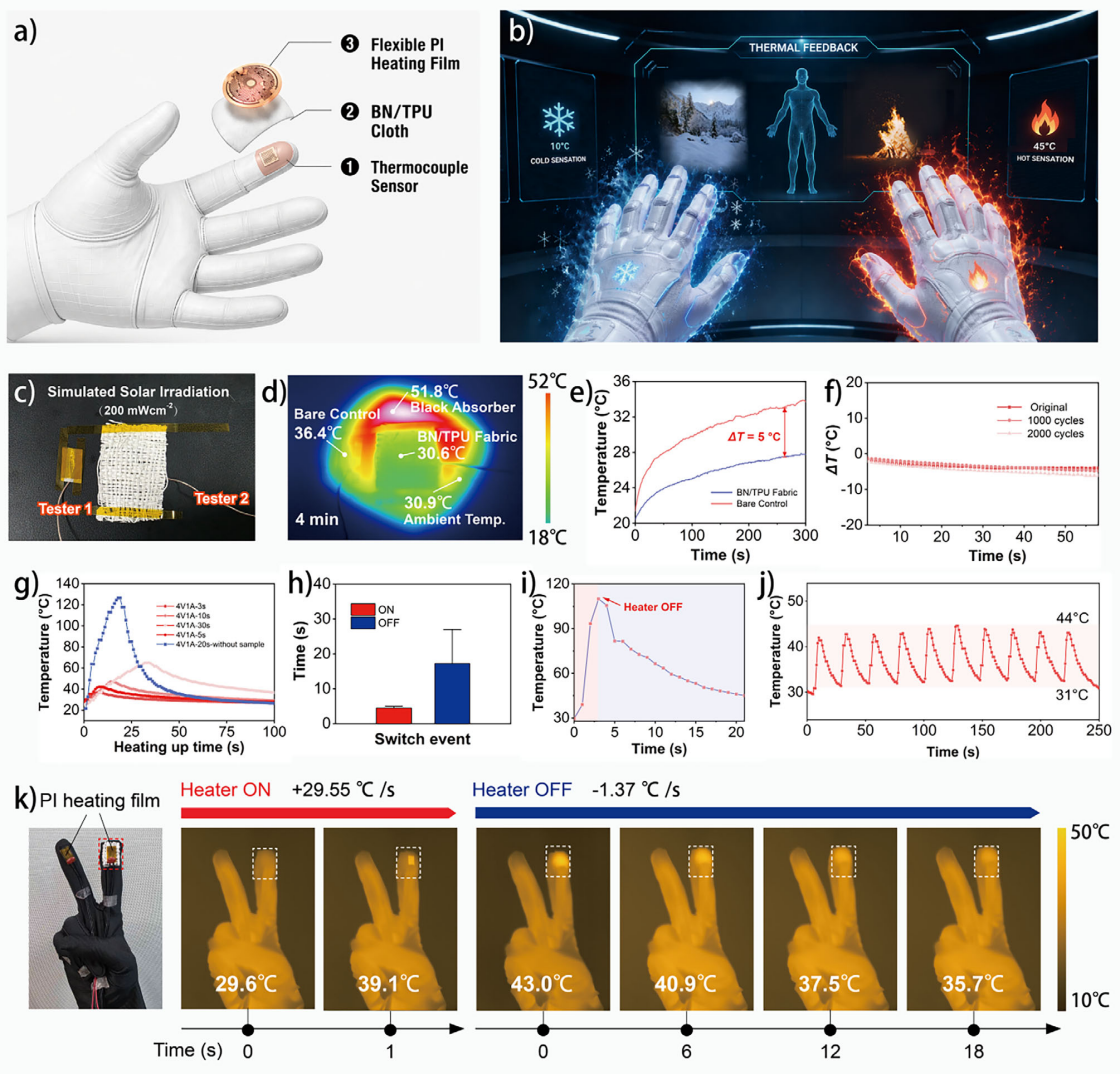
Passive cooling and thermal feedback performance of the h‐BN/TPU glove. (a) Exploded‐view illustration of the glove structure. (b) Schematic of the application scenario. (c–e) Passive thermal‐management performance under 200 mW·cm^−2^ simulated solar irradiation. (f) Thermal stability after 1000 and 2000 bending cycles. (g) Temperature profiles of the 30 wt.% h‐BN/TPU fabric and the bare control. (h) Response times of ON/OFF switching events. (i) Temperature evolution of the 30 wt.% h‐BN/TPU glove during 3 s heating. (j) Ten ON/OFF heating cycles. (k) Infrared image sequence during heating and cooling.

To evaluate passive thermal management, woven h‐BN/TPU fibers were exposed to 200 mW·cm^−2^ simulated solar irradiation. IR imaging revealed a 5.7°C reduction in surface temperature compared with bare TPU (Figure 4c–e), demonstrating effective heat spreading and thermal regulation. Thermal performance remained stable after 1000 and 2000 bending cycles (Figure 4f), confirming robust mechanical durability and thermal stability. These results establish bending tolerance as an encouraging first durability benchmark for wearable use; however, application‐level validation should also include cyclic tensile deformation, sweat/humidity exposure, interfacial friction, and washing‐related aging, because each may alter pore structure, inter‐fiber contact, or local thermal coupling during long‐term service. The hierarchical combination of rectangular fiber geometry, aligned h‐BN plates, controlled void fraction, and microporous matrix enables this coupled thermal‐mechanical performance.

For active thermal feedback, ON/OFF switching was applied using different pulse durations to match experimental goals: 3 s pulses for direct comparison with bare elastic fabric (Figure 4i), 2 s pulses for quantitative thermal cycling (Figure 4h,j), and 1 s short pulses for IR imaging to assess spatial confinement (Figure 4k). The glove exhibits a heating rate of +29.55°C·s^−1^ and a thermal relaxation time of ∼20 s (cooling from peak temperature to below the warm perception threshold of 35°C). IR testing curves confirm that the actuation region remains spatially confined, with lateral temperature decay consistent with targeted fingertip stimulation. Notably, while the glove was primarily tested in dry conditions, the humidity‐assisted enhancement observed at the fiber level suggests that perspiration‐induced local moisture may further reduce pore resistance and improve thermal coupling during extended wear, although this effect was not directly quantified at the device level in the present study. Furthermore, we have explored surface patterning of the h‐BN/TPU fibers via magnetron sputtering of copper, demonstrating the potential of these h‐BN/TPU fibers for integration into advanced flexible electronic devices and multifunctional wearable systems (Figure S12).

In summary, the 30 wt.% *h*‐BN/TPU fabric simultaneously functions as a passive thermal management layer that mitigates radiative heat gain (5.7°C reduction) and as an active thermal‐haptic substrate that delivers spatially confined fingertip stimulation—two functions previously achievable only with separate textiles. This dual‐function capability, combined with mechanical robustness and moisture‐assisted thermal transport, validates the fiber as a versatile platform bridging material design and device functionality for next‐generation thermally interactive wearables.

## 6 Conclusion

In conclusion, we have developed a highly stretchable 30 wt.% h‐BN/TPU fiber that unites anisotropic thermal conduction with excellent mechanical compliance (422% elongation at break). The combination of microfluidic spinning and non‐solvent‐induced phase separation produces aligned h‐BN plates within a hierarchical microporous TPU matrix, establishing continuous heat conduction paths while preserving flexibility. When assembled into a woven fabric, these fibers provide effective passive thermal management, reducing surface temperature by 5.7°C under simulated solar irradiation and maintaining performance after repeated bending cycles. Integrated into a thermal feedback glove, the h‐BN/TPU fabric enables spatially confined fingertip actuation with a heating rate of +29.55°C·s^−1^ and a thermal relaxation time of ∼20 s (below the warm perception threshold of 35°C), as verified by IR imaging. Moisture‐mediated thermal enhancement at the fiber level (4.14 W·m^−1^·K^−1^ under humid conditions) is expected to further improve thermal coupling during prolonged wear, primarily because absorbed water replaces insulating air within the pore network and thereby lowers pore‐associated thermal resistance. Notably, the microporous architecture plays a dual role across operating states: in the dry state it serves as a mechanical buffer that preserves elongation under filler‐induced stress, while under humid conditions the same pore network is filled by capillary‐uptaken water and may further assist local heat transfer between nearby h‐BN plates. Although the present study confirms good bending durability, future wearable qualification should further examine cyclic stretching, sweat and humidity aging, frictional abrasion, and washing stability to define the full service boundary of the fiber platform. Porosity is thus reframed not as a passive defect but as a multi‐state functional element of the dual‐mode design. By synergistically leveraging conductive and moisture‐assisted heat transport, this work demonstrates a versatile fiber‐based platform that bridges material design and device functionality, simultaneously supporting passive cooling and active haptic feedback, and providing a foundation for next‐generation thermally interactive wearable systems with broad applications in VR/AR, teleoperation, and adaptive personal thermal management.

## 7 Experimental Section

### 7.1 Materials

The *h*‐BN powders (57 µm) were provided by Shandong Jonye Advanced Material Co.,Ltd. The TPU particles were supplied by Wanhua Chemical Group Co.,Ltd. and the dimethylformamide (AR, 99.5%) was purchased from Shanghai Aladdin Biochemical Technology Co., Ltd.

### 7.2 Preparation of h‐BN/TPU Fibers

In the initial step, DMF was used to dissolve TPU particles at a 3:2 mass ratio. A 30 wt.% h‐BN/TPU solution was subsequently prepared by incorporating h‐BN powders into the TPU/DMF solution followed by homogenization using a speed mixer. The resulting h‐BN/TPU/DMF solution was then subjected to wet‐spinning to fabricate h‐BN/TPU composite fibers. During wet‐spinning, the h‐BN/TPU/DMF dope was extruded through a rectangular spinning channel with a width of 200 µm and an aspect ratio of 2.5; the injection velocity was varied from 0.1 to 1.2 mm min^−1^, and the collector rolling speed was varied from 20 to 68 s r^−1^ as the take‐up condition. For the optimized 30 wt.% fibers, the injection velocity was 0.3 mm min^−1^. The extruded fibers were coagulated in a water bath and then naturally dried for more than 24 h.

### 7.3 Characterization

The microstructural morphology of the samples was observed using a field emission scanning electron microscope (FE‐SEM, Quanta FEG 250, FEI, USA). The crystalline structure and surface chemical compositions were determined using x‐ray diffractometer (XRD, D8 Discover/GADDS, Bruker, Germany) with Cu Kα radiation, Wide‐Angle x‐ray Diffraction (WXRD, Xeuss 3.0 UHR, France), Raman spectrometer (Renishaw plc, Wotton‐under‐edger, UK) with a laser wavelength of 532 nm, Fourier transform infrared spectroscopy (FTIR, Nicolet 6700, Thermo Fisher Scientific, USA), respectively. The axial thermal diffusivity of bundled h‐BN/TPU fibers was measured by the laser flash method (LFA 467 NanoFlash, Netzsch, Germany), with the fibers aligned along the heat‐flow direction. Heat capacity was measured by differential scanning calorimetry (DSC, PYRIS Diamond, Perkin Elmer, USA), and the axial thermal conductivity was calculated as λ = α × ρ × Cp, where α, ρ, and Cp represent thermal diffusivity, density, and heat capacity, respectively. Thermogravimetric analysis of the composites and TPU matrix was conducted in the atmosphere at a heating rate of 10°C min^−1^ (TGA, TG‐209 F3, Netzsch, Germany). Thermal images were recorded by an infrared (IR) camera (Ti400, Fluke, USA). Single‐fiber tensile properties were measured using a 50 N universal testing machine (Zwick/Roell Z100) with an initial gauge length of 20 mm and a stretching rate of 10 mm s^−1^.

## Author Contributions

**Yiwei Zhou**: contributed to experimental design, data analysis, and manuscript preparation. **Shanshan Long**: conducted the fluid‐dynamics analysis. **Bo Chen**: conducted the weaving procedure and mechanical testing. **Maohua Li**, **Linhong Li**, **Yue Qin**, **Rongjie Yang**, **Hanxi Chen**, **Cheng‐Te Lin**, and **Nan Jiang**: contributed to discussion and manuscript revision. **Yixiang Xu**, **Kean How Cheah**, and **Jinhong Yu**: supervised the work, reviewed and revised the manuscript.

## Conflicts of Interest

The authors declare no conflicts of interest.

## Supporting information




**Supporting File**: advs78162‐sup‐0001‐SuppMat.docx.

## Data Availability

Data supporting the findings of this study are available from the corresponding authors upon reasonable request.
