# Supplementary material for: Stretchable h‐BN/TPU Fibers for Passive‐Active Dual‐Mode Thermal Management and Wearable Haptic Interfaces
Source: Adv Sci (Weinh). 2026 Oct 15:e78162. Online ahead of print. doi: 10.1002/advs.78162 (PMC13672248; doi:10.1002/advs.78162)
Supplement: Supplementary file 1 [file ADVS-9999-e78162-s001.docx]

**Supporting Information**

Stretchable h-BN/TPU Fibers for Passive-Active Dual-Mode Thermal Management and Wearable Haptic Interfaces

Yiwei Zhou, Shanshan Long, Bo Chen, Maohua Li, Linhong Li, Yue Qin, Rongjie Yang, Hanxi Chen, Cheng-Te Lin, Yixiang Xu*, Kean How Cheah*, Nan Jiang*, and Jinhong Yu*

Jinhong Yu: yujinhong@nimte.ac.cn; Yixiang Xu: [Yixiang.Xu@nottingham.edu.cn;](mailto:Yixiang.Xu@nottingham.edu.cn;) Kean How Cheah: [Kean-How.Cheah@nottingham.edu.cn;](mailto:Kean-How.Cheah@nottingham.edu.cn;) Nan Jiang: [jiangnan@nimte.ac.cn](mailto:yujinhong@nimte.ac.cn).

This Supporting Information includes additional figures, experimental notes, model details, and tables.

a)

b)

**Fig. S1.** Schematic illustration of particle migration and inertial focusing behavior in square and rectangular microchannels, drawn based on the inertial microfluidic principles reported by Amini et al.[S1]. a) Schematic showing the migration and alignment behavior of h-BN plates during injection through the rectangular microchannel. b) Hypothesized near-wall focusing locations of h-BN plates in the rectangular microchannel.

**Fig. S2. Dimensions of the rectangular microfluidic channel used for wet spinning.**

**Fig. S3. Large-diameter, highly crystalline h-BN plates were selected.** a) SEM image of representative large-diameter h-BN plates. b) Statistical analysis showing an average plate diameter of 57.34 µm. c) XRD pattern confirming the high crystallinity of the h-BN plates.

**Note S1.** Shear viscosity measurements were performed for h-BN/TPU fibers with varying filler contents (30-50 wt%). The experimental data were fitted using the Carreau model to quantify key rheological parameters, including zero-shear viscosity (*μ_₀_*), infinite-shear viscosity (*μ_∞_*), characteristic time constant (*λ*), and flow behavior index (*n*). The fitted curves capture the shear-thinning behavior of the composites and provide essential input for numerical simulations of the extrusion process and for optimizing processing conditions. The fitting curve is based on the Carreau model:

*μ = μ_∞_ + (μ_0_-μ_∞_) [1 + (λ·γ̇)²]^((n-1)/2)*	(1)

*μ*: shear viscosity

*μ_₀_*: zero-shear viscosity

*μ_∞_*: infinite-shear viscosity

*λ*: characteristic time constant

*γ̇*: shear rate

*n*: flow behavior index

The fitting curves are presented in Fig. S4.

**Fig. S4. The plot of viscosity as a function of shear rate and the fitting curve by the Carreau model.**

**Fig. S5. FEA results of injection process in both rectangular (a and b) and cylindrical (c) channels.**

**Note S2.**

To quantify the lattice orientation information of the h-BN/TPU fibers, we extended the diffraction peak azimuth to obtain the azimuth angle x (x=2*θ*) and analyzed the index of Hermans orientation factor (*f*) derived from WAXRD. Details of the analysis for index f are as follows.

$\text{f}\text{=}\frac{\text{1}}{\text{2}}\text{(3 }\left\langle\text{cos}^{\text{2}} \right.\left. \text{x} \right\rangle\text{−1)}$	(2)

$\left\langle\text{cos}^{\text{2}} \right.\left. \text{x} \right\rangle\text{=}\int_{\text{0}}^{\frac{\text{π}}{\text{2}}} {\text{(}\text{cos}}^{\text{2}}\text{x}\text{) }\text{dx}$	(3)

where *f* = 1.00 is perfect face-on orientation, *f* = - 0.5 is perfect edge-on orientation and *f* = 0 is a completely random, isotropic structure. Based on this, we found that for 30% h-BN/TPU composite, *f* was 0.72, suggesting that the excellent structural alignment of h-BN plates was achieved along the y-axis.

**Note S3.**

**Fig. S6. Schematic diagram of h-BN platelet alignment and the angle-resolved polarized Raman configuration.**

As illustrated in Fig. S6, a simplified model was used to describe h-BN platelet alignment and the angle-resolved polarized Raman configuration. The laboratory coordinate system (x, y, z) is indicated by black vectors, where the y-axis is aligned with the basal-plane direction of the 67 vol% h-BN/TPU composite and the x-axis is perpendicular to it. The laser propagates along the z direction, while the polarization angle θ relative to the basal plane is varied using a half-wave plate with a step size of 7.5°. The h-BN platelets were assumed to have a homogeneous orientation distribution with an average alignment angle α relative to the basal plane (y). The unit electric-field vectors of the incident laser and scattered Raman signal are denoted as eL and eS, respectively. Measurements were additionally collected at θ = 90° and 180°. The following equation describes the cumulative Raman intensity of the E2g mode contributed by the BN platelets:

IE2g (θ) = 1/2 c² cos^2^α{2 + cos[2(α-θ)] + cos[2(α+θ)]}	(4)

The E2g intensity reaches its maximum and minimum when the electric-field vector of the incident polarized laser eL is parallel (θ = 0° and 180°, IE2g (‖)) and perpendicular (θ = 90° and 270°, IE2g (⊥)) to the basal plane of the 67 vol% h-BN/TPU composite, respectively. The ratio IE2g (‖)/IE2g (⊥) equals cot²α and increases with decreasing α, where α is the alignment angle between the average BNNS orientation and the fiber-length direction (y), as shown in Fig. S6.

**Fig. S7. FEA results illustrate the heat transfer behavior of cylindrical and rectangular fibers along the x, y, and z axes.**

Fig. S8. FEA comparison of heat transfer in face-face and line-face contact modes. In the line-face contact model, representative of cylindrical fibers, the effective contact area between adjacent h-BN plates is limited. In contrast, the face-face contact mode in rectangular fibers provides larger planar overlap and faster heat transfer. Both models were heated at the hot end to 100 °C, and the cold-end temperature difference reaches approximately 10 °C at 0.25 s, indicating more rapid thermal equilibration in the face-face contact mode.

**Note S4.**

1. **Decoupled Single-Degree-of-Freedom Lewis-Nielsen Model**

This section presents the governing equations of the decoupled Lewis-Nielsen model:

$\text{k}_{\text{m}}^{\text{∗}}\text{=}\text{k}_{\text{TPU}}\text{⋅}\frac{\text{1+1.5}\text{B}_{\text{void}}\text{⋅}\text{ϕ}_{\text{void}}}{\text{1−}\text{B}_{\text{void}}\text{⋅}\text{ϕ}_{\text{void}}}$	(1)

$\text{B}_{\text{void}}\left( \text{ϕ}_{\text{BN}} \right)\text{=}\frac{\text{k}_{\text{void}}\text{/}\text{k}_{\text{TPU}}\text{−1}}{\text{k}_{\text{void}}\text{/}\text{k}_{\text{TPU}}\text{+1.5}}\text{⋅}\text{C}_{\text{void}}\left( \text{ϕ}_{\text{BN}} \right)$	(2)

$\text{ψ}_{\text{void}}\text{=1+}\frac{\text{1−}\text{ϕ}_{\text{m}}}{\text{ϕ}_{\text{m}}^{\text{2}}}\text{ϕ}_{\text{void}}$	(3)

$\text{C}_{\text{void}}\left( \text{ϕ}_{\text{BN}} \right)\text{=}\text{C}_{\text{low}}\text{⋅}\text{w}_{\text{low}}\text{+}\text{C}_{\text{h}\text{ig}\text{h}}\text{⋅}\text{w}_{\text{h}\text{ig}\text{h}}$	(4)

$\text{w}_{\text{low}}\text{=}\frac{\text{ϕ}_{\text{c}}}{\text{ϕ}_{\text{BN}}\text{+}\text{ϕ}_{\text{c}}}\text{, }\text{w}_{\text{h}\text{ig}\text{h}}\text{=}\frac{\text{ϕ}_{\text{BN}}}{\text{ϕ}_{\text{BN}}\text{+}\text{ϕ}_{\text{c}}}$	(5)

**Parameter definitions:**

| **Parameter** | **Physical meaning** | **Value** |
| --- | --- | --- |
| $\text{k}_{\text{TPU}}$ | Thermal conductivity of TPU matrix | 0.3 W·m^-1·^K-1 |
| $\text{k}_{\text{BN}}$ | Thermal conductivity of BN filler | 200 Wm^-1^K^-1^ |
| $\text{k}_{\text{void}}$ | Thermal conductivity of air-filled pores | 0.025 Wm^-1^K^-1^ |
| $\text{φ}_{\text{BN}}$ | Volume fraction of BN | See sample table |
| $\text{φ}_{\text{void}}$ | Volume fraction of pores | See sample table |
| $\text{φ}_{\text{m}}$ | Maximum packing fraction of BN | 0.736 |
| $\text{C}_{\text{low}}$ | Pore suppression factor at low BN content | 3.075 (fitted) |
| $\text{C}_{\text{high}}$ | Pore suppression factor at high BN content | 1.0 (physical limit) |
| $\text{φ}_{\text{c}}$ | Threshold separating isolated and network regimes | 0.12 |
| $\text{w}_{\text{low}}\text{,}\text{ }\text{w}_{\text{high}}$ | Weighting factors for low/high BN content | Calculated from formula |
| $\text{k}_{\text{m}}^{\text{∗}}$ | Corrected matrix conductivity (including pore effect) | Computed value |
| $\text{B}_{\text{void}}$ | Pore depolarization factor | Computed value |
| $\text{ψ}_{\text{void}}$ | Pore packing correction factor | Computed value |
| $\text{C}_{\text{void}}\left( \text{ϕ}_{\text{BN}} \right)$ | Effective pore resistance coefficient | Computed value |

This model allows explicit treatment of pore suppression effects across low and high BN fractions, providing a clear connection between microstructural characteristics and macroscopic thermal conductivity.

2. **Fitted results**

**Table S1.** **Composition, measured properties, Lewis-Nielsen fit results, and per-filler efficiency of the four h-BN/TPU fiber samples.**

*Δk/*$\text{k}_{\text{exp}}$*= (*$\text{k}_{\text{fit}}$*−*$\text{k}_{\text{exp}}$*)/*$\text{k}_{\text{exp}}$ expressed as percentage; positive values indicate model overestimation. $\text{k}_{\text{exp}}$/$\text{φ}_{\text{BN}}$ is the per-unit-filler thermal conductivity, a measure of filler efficiency.

| Sample | $\text{φ}_{\text{BN}}$ | $\text{φ}_{\text{void}}$ | $\text{k}_{\text{exp}}$  (Wm⁻¹K⁻¹) | $\text{k}_{\text{fit}}$  (Wm⁻¹K⁻¹) | $\text{k}_{\text{exp}}$*/*$\text{φ}_{\text{BN}}$  (Wm⁻¹K⁻¹) | *ε*  (%) |
| --- | --- | --- | --- | --- | --- | --- |
| 1 | 0.087 | 0.250 | 0.53 | 0.857 | 6.1 | 495 |
| 2 | 0.118 | 0.064 | 2.08 | 2.697 | 17.6 | 423 |
| 3 | 0.159 | 0.205 | 2.47 | 2.369 | 15.5 | 139 |
| 4 | 0.210 | 0.247 | 3.29 | 3.036 | 15.7 | 109 |

* Fitted parameter: $\text{C}_{\text{low}}$=3.075, $\text{C}_{\text{high}}$=1.0. The reported *R^2^* = 0.986 is calculated only for the filler-network regime samples S2-S4 in the parity plot; S1 is treated as an island-regime reference outside the model domain.

$\text{k}_{\text{exp}}\text{k}_{\text{fit}}\text{k}\text{φ}_{\text{BN}}\text{φ}_{\text{BN}}\text{φ}_{\text{m}}\text{φ}_{\text{BN}}\text{φ}_{\text{m}}\text{φ}_{\text{void}}\text{C}_{\text{void}}\text{φ}_{\text{BN}}\text{C}_{\text{h}\text{ig}\text{h}}\text{ }$Fig. S9. Predicted and experimental thermal conductivity and pore-suppression analysis of h-BN/TPU composite fibers. (a) Parity plot for the filler-network-regime samples S2-S4, where R² = 0.986 is calculated after excluding the island-regime reference sample S1. Dashed line represents y = x and dotted lines indicate ±10% deviation. (b) Model-predicted thermal conductivity as a function of BN volume fraction for different void fractions, with experimental points S1-S4 overlaid. (c) Fitted pore-suppression factor as a function of BN content with the 95% confidence interval from Monte Carlo simulations; S1 is shown as the extrapolated island-regime reference, and the horizontal dashed line indicates the spherical-limit value of 1.

3. **SEM microstructure verification**

**Table S2.** Macro- and Mesopore Dimensions in 30 wt% h-BN/TPU Composite Fibers. (SEM Analysis)

| No. | Length (μm) | Width (μm) | Lateral size |
| --- | --- | --- | --- |
| 1 | 8.239 | 6.161 | 1.337282909 |
| 2 | 8.635 | 6.795 | 1.270787344 |
| 3 | 2.185 | 2.107 | 1.037019459 |
| 4 | 1.752 | 1.455 | 1.204123711 |
| 5 | 3.53 | 1.827 | 1.932129174 |
| 6 | 1.948 | 1.314 | 1.482496195 |
| 7 | 3.412 | 1.469 | 2.322668482 |
| 8 | 1.532 | 1.276 | 1.200626959 |
| 9 | 1.029 | 0.959 | 1.072992701 |
| 10 | 2.046 | 1.276 | 1.603448276 |
| 11 | 2.624 | 1.404 | 1.868945869 |
| 12 | 2.679 | 2.058 | 1.301749271 |
| 13 | 2.654 | 1.48 | 1.793243243 |
| 14 | 2.581 | 2.312 | 1.116349481 |
| 15 | 1.102 | 1.087 | 1.013799448 |
| 16 | 2.745 | 1.988 | 1.380784708 |
| 17 | 1.469 | 1.276 | 1.151253918 |
| 18 | 2.369 | 2.203 | 1.075351793 |
| 19 | 1.579 | 1.228 | 1.285830619 |
| 20 | 1.814 | 0.92 | 1.97173913 |
| 21 | 1.998 | 1.009 | 1.980178394 |
| 22 | 2.153 | 1.378 | 1.562409289 |
| 23 | 1.354 | 1.314 | 1.0304414 |
| 24 | 1.502 | 1.021 | 1.471106758 |
| 25 | 1.48 | 1.228 | 1.205211726 |
| 26 | 2.884 | 1.513 | 1.906146728 |
| 27 | 1.469 | 1.276 | 1.151253918 |
| 28 | 1.627 | 1.276 | 1.27507837 |
| 29 | 1.912 | 0.942 | 2.029723992 |
| 30 | 0.766 | 0.641 | 1.1950078 |

* L/W$\text{≈}$1.44

4. **Monte Carlo Uncertainty Analysis**

Monte Carlo analysis confirms the robustness and decoupling of fitted parameters.

- N = 1000 iterations
- $\text{C}_{\text{low}\text{:}\text{medium}}$ = 3.075, 95% CI = [3.0, 3.15]
- $\text{C}_{\text{high}\text{:}\text{medium}}$ = 1.0, 95% CI = [1.0, 1.0]
- *r(*$\text{C}_{\text{low}}$*/*$\text{C}_{\text{high}}$*)* = -0.204

**Note S5.** **Random-forest permutation-importance methodology.**

We note that, owing to the modest dataset size, permutation importance was evaluated on the training set rather than a held-out validation set; the reported importance values should therefore be interpreted as reflecting in-sample variable sensitivity rather than out-of-sample predictive contribution. The random-forest permutation-importance analysis used in Fig. 3i was implemented with scikit-learn 1.8.0, using 63 fiber samples spanning the processing-parameter ranges of Fig. 3g–h.[S2] The model used 300 trees with default max depth, trained on 100% of the dataset with 20 permutations per feature for importance estimation. Importance values reported in Fig. 3i are mean ± standard deviation across permutations.

Fig. S10. Photographs of the 30 wt% h-BN/TPU fabric after 2000 bending cycles.

Fig. S11. Linear fitting of the temperature profiles of the h-BN/TPU glove.

Fig. S12. SEM images of the surface-patterned 30 wt% h-BN/TPU fiber.

**References**

[S1] H. Amini, W. Lee, D. Di Carlo, *Lab Chip* **2014**, *14*, 2739.

[S2] F. Pedregosa, G. Varoquaux, A. Gramfort, V. Michel, B. Thirion, O. Grisel, M. Blondel, P. Prettenhofer, R. Weiss, V. Dubourg, J. Vanderplas, A. Passos, D. Cournapeau, M. Brucher, M. Perrot, E. Duchesnay, *J. Mach. Learn. Res.* **2011**, *12*, 2825-2830.
